# Supplementary material for: Causal Relationship between Aging and Anorexia Nervosa: A White-Matter-Microstructure-Mediated Mendelian Randomization Analysis
Source: Biomedicines. 2024 Aug 16;12(8):1874. doi: 10.3390/biomedicines12081874 (PMC11351342; doi:10.3390/biomedicines12081874)
Supplement: Supplementary file 1 [file biomedicines-12-01874-s001.zip › Supplementary Figure/Figure S4/Singe SNP plot of MR analysis for TL to ubm-b-1462.pdf]

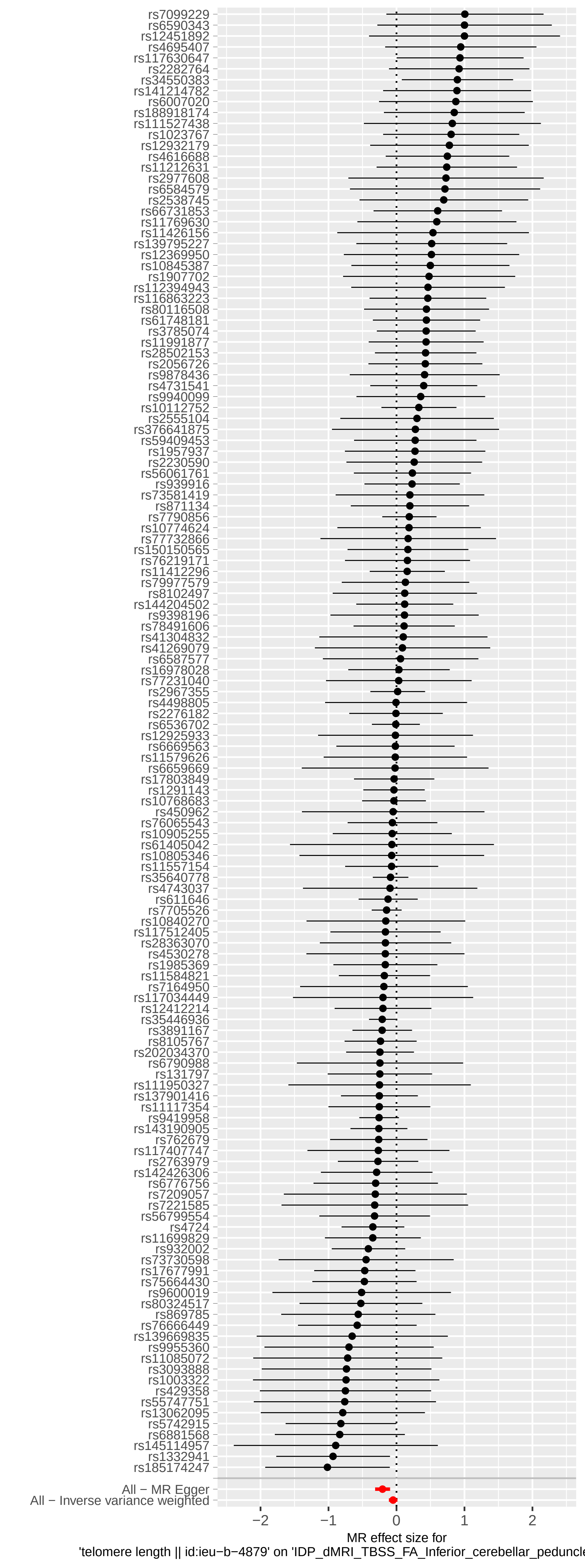
rs7099229
rs6590343
rs12451892
rs4695407
rs117630647
rs2282764
rs34550383
rs141214782
rs6007020
rs188918174
rs111527438
rs1023767
rs12932179
rs4616688
rs11212631
rs2977608
rs6584579
rs2538745
rs66731853
rs11769630
rs11426156
rs139795227
rs12369950
rs10845387
rs1907702
rs112394943
rs116863223
rs80116508
rs61748181
rs3785074
rs11991877
rs28502153
rs2056726
rs9878436
rs4731541
rs9940099
rs10112752
rs2555104
rs376641875
rs59409453
rs1957937
rs2230590
rs56061761
rs939916
rs73581419
rs871134
rs7790856
rs10774624
rs77732866
rs150150565
rs76219171
rs11412296
rs79977579
rs8102497
rs144204502
rs9398196
rs78491606
rs41304832
rs41269079
rs6587577
rs16978028
rs77231040
rs2967355
rs4498805
rs2276182
rs6536702
rs12925933
rs6669563
rs11579626
rs6659669
rs17803849
rs1291143
rs10768683
rs450962
rs76065543
rs10905255
rs61405042
rs10805346
rs11557154
rs35640778
rs4743037
rs611646
rs7705526
rs10840270
rs117512405
rs28363070
rs4530278
rs1985369
rs11584821
rs7164950
rs117034449
rs12412214
rs35446936
rs3891167
rs8105767
rs202034370
rs6790988
rs131797
rs111950327
rs137901416
rs11117354
rs9419958
rs143190905
rs762679
rs117407747
rs2763979
rs142426306
rs6776756
rs7209057
rs7221585
rs56799554
rs4724
rs11699829
rs932002
rs73730598
rs17677991
rs75664430
rs9600019
rs80324517
rs869785
rs76666449
rs139669835
rs9955360
rs11085072
rs3093888
rs1003322
rs429358
rs55747751
rs13062095
rs5742915
rs6881568
rs145114957
rs1332941
rs185174247
All − MR Egger
All − Inverse variance weighted
−2
−1
0
1
2
MR effect size for
'telomere length || id:ieu−b−4879' on 'IDP_dMRI_TBSS_FA_Inferior_cerebellar_peduncle
